# First-year follow-up of children with chronic nonbacterial osteomyelitis—an analysis of the German National Pediatric Rheumatologic Database from 2009 to 2018

**DOI:** 10.1186/s13075-021-02658-w

**Published:** 2021-11-08

**Authors:** Christiane Reiser, Jens Klotsche, Anton Hospach, Rainer Berendes, Anja Schnabel, Annette F. Jansson, Markus Hufnagel, Nadine Grösch, Martina Niewerth, Kirsten Minden, Hermann Girschick

**Affiliations:** 1 Pediatric Rheumatology, Department of Pediatrics, Landeskrankenhaus Bregenz, Bregenz, Austria; 2 German Rheumatism Research Center, a Leibniz Institute, Berlin, Germany; 3 Charité – Universitätsmedizin Berlin, corporate member of Freie Universität Berlin, Humboldt-Universität zu Berlin, and Berlin Institute of Health, Berlin, Germany; 4 Department of Pediatrics, Olgahospital, Klinikum Stuttgart, Stuttgart, Germany; 5 Pediatric Rheumatology, Children’s Hospital St. Marien, Landshut, Germany; 6 Children’s Hospital, Gustav Carus University, Dresden, Germany; 7 Department of Pediatrics, Dr von Hauner Children’s Hospital, Ludwig Maximilian University, Munich, Germany; 8 Division of Pediatric Infectious Diseases and Rheumatology, Department of Pediatrics and Adolescent Medicine, University Medical Center, Medical Faculty, University of Freiburg, Freiburg, Germany; 9 Vivantes Klinikum Friedrichshain, Children’s Hospital, Berlin, Germany; 10 Children’s Hospital, University of Wuerzburg, Wuerzburg, Germany; 11 German Center for Growth and Development “DEUZWEG”, Berlin, Germany

**Keywords:** Chronic nonbacterial osteomyelitis, Pediatric rheumatology, Autoinflammation, Chronic nonbacterial multifocal osteomyelitis

## Abstract

**Objective:**

To assess the first-year features of patients with chronic nonbacterial osteomyelitis (CNO).

**Methods:**

Patients with a diagnosis of CNO, disease duration of under 13 months, and first registration in the German National Pediatric Rheumatologic Database (NPRD) between 2009 and 2018 were included in this cross-sectional analysis.

**Results:**

Of 774 documented patients, 62.8% were female, and all patients had a median age of 11 years. The most affected clinical sites were the tibia (29.7%), pelvis (28.0%), and femur (27.8%). HLA-B27 was positive in 48 of 314 analyzed patients (15.3%). In 406 patients, an X-ray was performed at the first visit; X-ray results showed osteosclerosis/−lysis in 34.0% and hyperostosis in 14.5% of the patients. MRI scans (focal and whole-body scans) were performed in 648 patients, and 81.5% showed a positive TIRM/STIR signal. A total of 84.7% of the patients were administered nonsteroidal anti-inflammatory drugs, 9.6% were administered oral glucocorticoids, 10.8% were administered disease-modifying anti-rheumatic drugs (DMARDs), and 6.1% were administered bisphosphonates. An evaluation of the patient’s questionnaire showed an overall well-being (NRS 0–10) of 2.0. The PedCNO disease “activity” score revealed a 70% improvement in variables in 43% of patients in the initial 1-year follow-up. Copresentation with diagnostic criteria of pediatric enthesitis-related arthritis was rare.

**Conclusion:**

To our knowledge, the NPRD cohort seemed to be the largest cohort of children and adolescents suffering from CNO worldwide. Most patients were treated effectively with NSAIDs, and only a small group of patients was administered additional medication. The patient-defined measures of disease activity had a moderate impact on patients’ daily lives.

**Trial registration:**

Not applicable.

**Supplementary Information:**

The online version contains supplementary material available at 10.1186/s13075-021-02658-w.

## Background

Chronic nonbacterial osteomyelitis (CNO) is an autoinflammatory disease of the bone of unknown etiology. The most severe and/or recurrent form of CNO is referred to as chronic recurrent multifocal osteomyelitis (CRMO) [1]. CNO affects the metaphyses of the long bones, but inflammation can be found in the patient’s whole skeleton with the exception of the neurocranium [2]. The general condition and quality of life in most children with this ailment seems to be reasonably good [3]. However, even unifocal lesions may elicit significant pain and may be debilitating. CNO is a multisystemic disease, and organs other than the bone and joints, predominately the skin with psoriasiform or pustular eruptions and the intestines with chronic inflammatory bowel diseases, can also be affected [2, 4]. Overlap forms with and evolution of CNO into other rheumatic diseases, such as SAPHO (synovitis, acne, pustulosis, hyperostosis, osteitis) syndrome (supposed adult variant of CNO) and/or enthesitis-related juvenile idiopathic arthritis (ERA), have been documented [5–7]. Confirming the diagnosis of CNO can be challenging, as the list of differential diagnoses is long and no specific laboratory markers exist thus far. Routine laboratory analysis usually reveals normal or moderately elevated inflammatory markers. The distinction of a possible bacterial infection or malignancy can be challenging [8]. Two diagnostic scores have been developed, though they have not been completely validated [9, 10]. International efforts to improve classification, diagnosis, and treatment are on the way and may support clinical diagnosis and care in the future [11].

The radiological approach varies from conventional radiographics and scintigraphy to the currently preferred MRI (T2 fat suppression sequences, TIRM/STIR, whole-body technique), which already reveals the bone edema in early stages of the disease [12].

First-line treatments are nonsteroidal antirheumatic drugs (NSAIDs), which can lead to inactive disease [13–15]. Depending on the course and severity, other drugs, such as steroids, methotrexate, bisphosphonates, or biologicals, have been used successfully in NSAID refractory patients [16]. In 2018, international Consensus Treatment Plans (CTPs) were published pointing out treatment strategies for NSAID-refractory patients [11].

The objective of this study was to show demographic, clinical, imaging, and treatment data of 774 patients at diagnosis onset (disease course ≤1 year) enrolled in the German National Pediatric Rheumatologic Database (NPRD) from 2009 to 2018. Here, we report the diagnostic and therapeutic parameters of these patients at disease onset, at first documentation in the registry and during the first year of follow-up.

## Patients and methods

### Patients

A wide range of patients with juvenile rheumatic diseases is included in the National Pediatric Rheumatologic Database. On a yearly basis, patients (or their parents) and pediatric rheumatologists documented sociodemographic and clinical parameters via standardized questionnaires. More than 60 pediatric rheumatology centers in Germany and Austria participated in NPRD and contributed patients with CNO for this study (see list in the appendix). The German registry covers more than half of all patients with inflammatory rheumatic diseases in Germany and provides representative data in terms of clinical and sociodemographic features, treatment modalities, and outcomes [17].

A CNO-specific questionnaire for the registry was developed in 2009. Over 10 years until 2018, we evaluated different features of CNO, including the following items: demographic, clinical and laboratory parameters (ESR, CrP, HLA-B27), biopsy (histological and microbiological results), duration and activity of the disease, comanifestations, and radiological diagnostics (conventional X-ray and/or MRI). A differentiation of the MRI mode used (whole-body versus focal) was established in the registry in 2016. Furthermore, course of disease—reach of inactive disease—, treatment modalities, the PedCNO score (a composite “treatment” score) [18], patient-reported overall well-being, and physician-reported disease severity (both assessed on a numeric rating scale NRS: 0 = inactive disease, 10 = highly active disease) were analyzed. Patients ≥13 years of age and/or the parents of affected younger children initially and thereafter reported functional abilities via the German version of the Childhood Health Assessment Questionnaire (C-HAQ) [19]. The resulting score ranges from 0 to 3 (0 = no functional disabilities, 3 = severe disability/unable to perform the activity) [20]. The PedCNO score consists of five core variables: ESR, number of radiological lesions, severity of disease estimated by physician/patient, and C-HAQ. Out of the five variables, score categories of 30%, 50%, and 70% improvement were calculated. For example, a PedCNO30 score implies a 30% improvement in at least three out of five core set variables, with no more than one parameter deteriorating by 30%. During follow-up, the clinical as well as the radiological number and localization of the affected bones was noted, including the type of imaging modality. In addition, factors presumably associated with disease activity (hyperostosis, fractures, peripheral arthritis, sacroiliitis, and skin lesions including acne, psoriasis, palmoplantar pustulosis, and undefined pustulosis; histology of bone biopsies (lymphocytic, granulocytic, fibrotic)) were analyzed. During the 10-year recruitment period, in addition to the analysis of the overall cohort, the dataset was divided into three time periods—A (2009–2012), B (2013–2015), and C (2016–2018)—to detect changes in diagnostic or therapeutic approaches over time. We have anticipated, based on newly available knowledge on CNO as an entity, that inclusion characteristics, CNO management (such as biopsies) and therapeutic strategies would change over time.

Cumulative treatment that had been administered prior to the first documentation inside the registry was reported in detail.

### Criteria applied for inclusion of the patients in the cross-sectional analysis

Since no validated diagnostic criteria for CNO/CRMO exist thus far, an expert-“confirmed” diagnosis of nonbacterial osteomyelitis was the basis for enrollment in the database. Patients who were recorded for the first time within the 10-year period and who clinically had symptomatic uni- and multifocal inflammatory bone lesions were included. There had to be an exclusion of a bacterial origin of disease, either in blood analysis or microbial biopsy analysis. Data from individual patients were reviewed for inclusion by three of the authors (CR, JK, and HG). Two diagnostic scores, which had been developed in the literature thus far, served as a basis for developing the CNO questionnaire in the registry [9, 10]. Patients with a disease duration of < 13 months until first documentation in the registry were considered for inclusion in this current analysis. In differential diagnosis, bacterial osteomyelitis and bone tumors were ruled out by biopsy and imaging. In addition, we asked for rheumatologic comanifestations of enthesitis-related arthritis, arthritis, sacroiliitis, and psoriatic arthritis.

### Statistical analysis

Continuously distributed data were reported by means, standard deviations and medians, and categorical data were reported by absolute and relative frequencies. Characteristics of groups of patients, e.g., HLA-B27-positive versus HLA-B27-negative patients, were compared by the Mann-Whitney *U* test and chi^2^ tests as appropriate. The PedCNO score was reported for patients with follow-up documentation available 1 year after first inclusion in NPRD.

## Results

Starting in 2009, 1675 patients were included in the registry. Of those, 774 patients had a disease duration of less than 13 months and were enrolled in this study. The gender distribution showed 486 (62.8%) female and 288 male patients. The median age at disease onset was 11 years (standard deviation STD 3.1) (additional file 1). The age ranged from 1 to 18 years. Of note, in the three different time periods (A: 2009–2012, B: 2013–2015, C: 2016–2018), no change was noted in the age or gender distribution (Table 1).

**Table 1:** Distribution of age and gender in the whole cohort and different time periods

	Total cohort	A (2009–2012)	B (2013–2015)	C (2016–2018)
Gender (female)	62.8%	102 (67.6%)	158 (60.1%)	226 (62.8%)
Median age (years)	11.0	12.0	11.0	11.0

We did not find changes in the duration from disease onset until first contact with a pediatric rheumatologist (A: mean 3.7 months, B: 4.0, C: 4.0). The median duration of disease until documentation into the registry varied over time (9.2, 8.2, and 8.0 months, respectively, *p* = 0.032).

### Clinical disease severity

The *initial disease activity* at presentation was reported by the physician with a mean of 2.1 on the NRS. The evaluation of the patient’s questionnaire showed pain NRS with a mean of 2.6, general well-being with a mean of 2.5, and C-HAQ with a mean of 0.28 at the first documented registry visit. In the three different time periods, these three parameters worsened over time. The *initial disease activity* was reported to be 1.9 (A), with 2.0 (B), and 2.2 (C) (*p* = 0.29). The patients’ noted *pain* was reported with a mean of 2.0 in A, 2.4 in B, and 2.9 in C (*p* = 0.0009). The patients' noted *general well-being* with a mean of 2.3 in A, 2.0 in B, and 2.8 in C (*p* = 0.014); thus, patients started in the registry with a slight increase in disease activity over time. The discrepancy between the doctor's and patient's assessments may reflect increased expectations and anxiety of the parents over the 10 year period.

### Musculoskeletal involvement

**Fig. 1:**
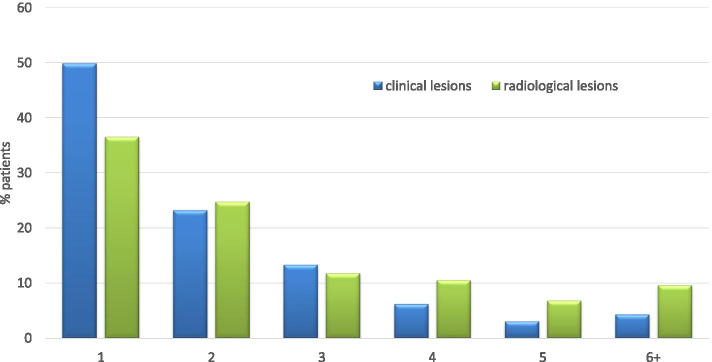
Number of clinical and radiological lesions (% patients). *N* = 323 patients with documented clinical **and** radiological lesions

**Fig. 2:**
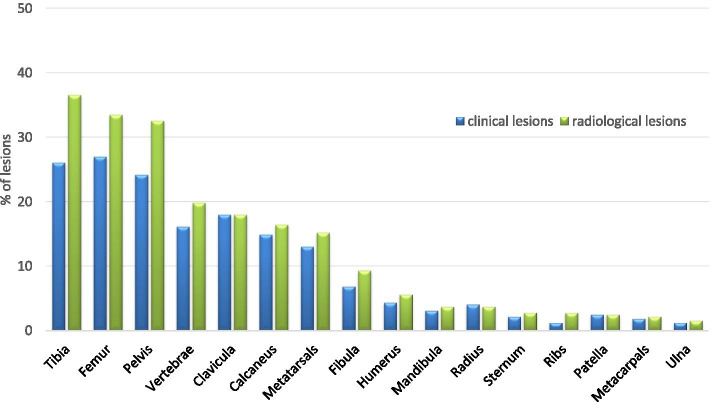
Clinically and radiologically defined location of lesions in %. *N* = 323 patients with clinical and radiological lesions

In 589 of 774 patients, a detailed description of the lesional location was documented. Clinically, 48.2% (*n* = 284) had a unifocal bone lesion, and 10.2% had five or more bone lesions. In 589 patients, the clinically most frequently affected sites were the tibia (29.7%), pelvis (28.0%), and femur (27.8%). Ninety-six individuals (16.3%) indicated that their spines were affected, 14.8% patients showed lesions in the metatarsal bones, and 13.9% patients showed lesions in the calcaneus. In most patients, MRI-defined active lesions corresponded to the clinical sites (Figs. 1 and 2).

However, by MRI, more lesions were identified in the pelvis, femur, and tibia compared to the clinical notification, showing that MRI reveals a higher sensitivity in diagnosing bone lesions than clinical judgment (Fig. 2).

In addition, a higher number of bone lesions per patient (four or more) were identified by MRI (*p* < 0.001).

Aside from bone pain, the following symptoms at first presentation were described: local erythema (3.9%, *n* = 593), any bone lesion in 89 of 774 patients (11.5%), predefined as pathological fractures in 7 (0.9%), vertebral fractures in 15 (1.9%), and hyperostosis in 36 patients (4.7%). MRI-defined vertebral fractures changed/increased at inclusion from A: 0.7% to B: 2.3% and C: 2.5% of patients; however, the difference was not statistically significant (*p* = 0.3).

Clinical signs of arthritis were noted in 179 of 732 patients (24.5%); these signs were located in the peripheral joints in 126 patients (17.2%), and 4.8% of the patients were found to have sacroiliitis. In 2.4% of patients, the reporting physician confirmed the diagnosis of enthesitis-related arthritis (ERA) based on the revised ILAR criteria of 2004 (*N* = 16 of 672) [21]. One percent of patients were diagnosed with psoriatic arthritis. Over time, initial clinical concomitant diagnosis of any arthritic manifestation was reported less commonly (A: 33.1%; B: 25.2%; C: 20.9% of patients, *p* = 0.103). This impression by the treating physician was supported by MRI-defined arthritis (TIRM/STIR imaging-defined synovitis and/or gadolinium uptake in the synovia), which changed from A: 14.5% to B: 8.1% and C: none (*p* < 0.001). Sacroiliitis was noted in A: 5.6%, B: 2.8%, and C: 5.9% of patients, *p* = 0.193.

### Involvement of other organs and growth characteristics

**Fig. 3:**
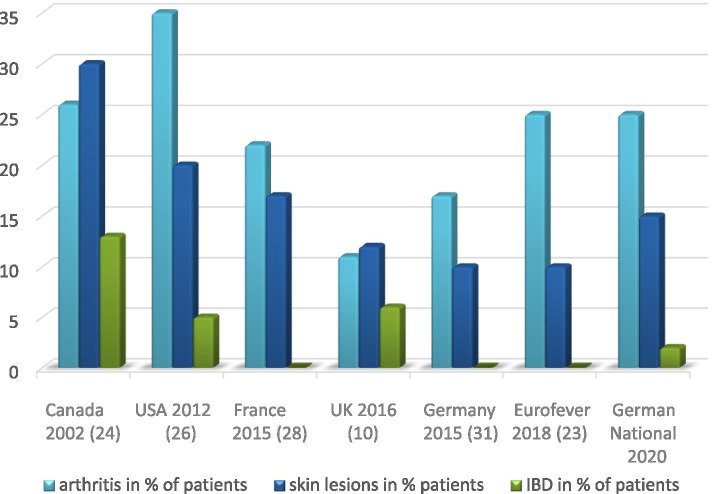
Concomitant clinical features in different CNO cohorts. IBD inflammatory bowel disease

Involvement of the skin was reported in 14.8% (112/757) of the patients: 3.6% had a diagnosis of psoriasis, 4.8% showed palmoplantar pustulosis, 4.1% showed acne, and 1.3% had undifferentiated pustules. A total of 2.5% of patients had no further delineated skin involvement. Over time, the presence of initial palmoplantar pustulosis decreased (A: 8.1%, B: 3.5%, and C: 4.3%, *p* = 0.098). Chronic inflammatory bowel disease overall was present in 12/756 patients (1.6%) (Fig. 3).

Initial concomitant diagnosis of IBD was less commonly noted in the further course (A: 2.7%, B: 2.3%, and C: 0.6%, *p* = 0.103).

**Fig. 4:**
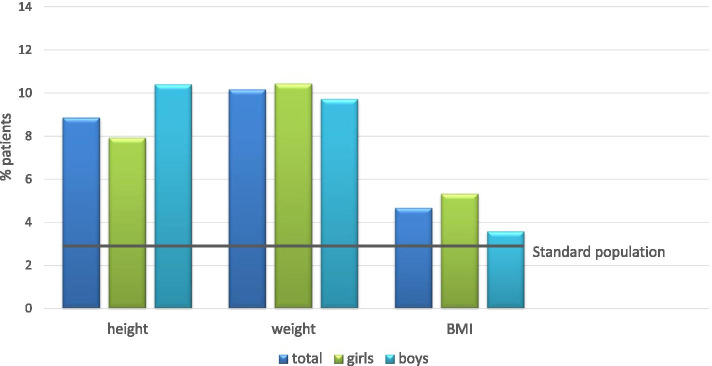
Gender distribution of parameters length, weight, and body mass index below the 3rd percentile at diagnosis. BMI body mass index

Since the age range was wide from 1 to 18 years, we calculated length, weight, and BMI in relation to a German age-matched reference cohort [22]: body length was 0.48 SDS, bodyweight 0.47 SDS, and body mass index (BMI) 0.09 SDS each below the means of this reference data set. In the subgroup analysis of patients with codiagnosis of CNO *and* IBD (*N* = 12), the body length was 0.87 SDS, bodyweight 0.96 SDS, and BMI 0.39 SDS, each even further below the means of this reference data set. The number of patients with length, weight, and body mass index below the third percentile was higher than in the reference cohort (Fig. 4).

**Fig. 5:**
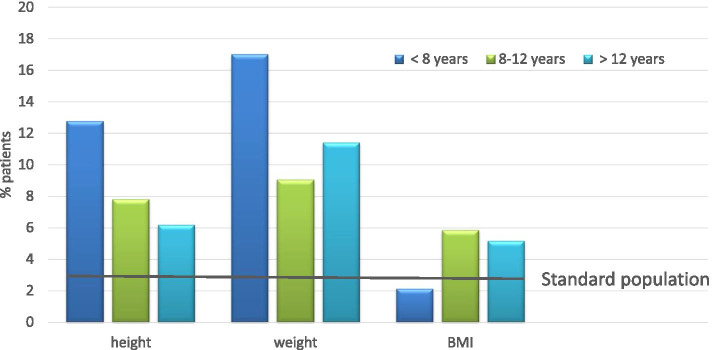
Age distribution of parameters length, weight, and body mass index below the 3rd percentile at diagnosis. BMI body mass index

Patients below the age of eight had particularly lower results for length and weight. Height and weight were significantly lower in all age groups (below 8, 8–12, above 12) than in the reference cohort (Fig. 5).

Over the time periods A/B/C, no significant changes were noted in height, weight, or BMI, still being below the average at the time of initial documentation.

Aside from musculoskeletal complaints, initial fever (> 38 °C) was noted in 77/593 patients (13.0%).

### Laboratory tests

An elevated CRP > 1 mg/dl was noted in 107/593 patients (18.0%). The mean ESR (based on 491 analyses) was 18.7 mm per hour. Over the three time periods, fewer biopsies were reported (A: 69.1%, B: 49.4%, C: 54.8%). Microbial analysis of biopsies was reported in 45% of the patients, almost all of them using tissue culture (> 90%). This pattern of analysis did not change over time (A-B-C). By definition, cultures had to be negative. In 11% of patients, negative cultures were supported by 16S rRNA universal PCR testing for eubacterial genes; over time, the frequency of PCR testing increased significantly from A: 4.1%, to B: 10.8%, and to C: 14.9%; *p* = 0.023. In addition, mycobacterial PCR was performed in 12% of patients, also increasing over time from A: 8.3%, B: 9.6%, to C: 15.4%; *p* = 0.15.

### Musculoskeletal imaging

In 406 patients, X-rays were performed during the time before the first documentation, showing spongiosal osteosclerosis/−lysis in 34% and periosteal hyperostosis in 14.5% of the patients. Vertebral fractures were reported in 36 patients (4.7%). In 177/406 patients, no changes were detected in conventional X-rays. In the 10-year recruitment period, the number of X-rays performed increased from 48% (A) to 65% (C). This tendency can also be observed in the MRI scans, where we see an increase over the years from 77% (A) to 84% (B) and 86.4% (C). MRI scans were performed in 648 patients, 81.5% of which showed a positive T2/TIRM/STIR signal, and 55.3% of which revealed relevant gadolinium contrast media uptake in the lesions. Adjacent soft tissue involvement (tissue edema, myositis) was seen in 161 (24.9%) patients. Of interest, arthritis was noted in 35 individuals (5.4%) by MRI. The main radiological (X-rays and MRI) bone lesion locations were noted in the tibia, pelvis, and femur (36.5%, 32.5%, and 31.2%, respectively (*n* = 378)). By radiological imaging, 37.3% of patients had a unifocal lesion, and 8.7% had six lesions or more (Fig. 1). Of interest, only 4.2% (*n* = 27) of patients were considered negative in MRI analysis, but 43% (*n* = 177, *p* < 0.001) were considered negative in X-rays. We took a close look at those 27 patients in the pre-analysis due to the MRI findings reported as negative. The initial questionnaire used did not distinguish between whole body MRI and focal MRI. We assume that some patients underwent focal MRI that was negative, while other investigations—e.g., X-ray or bone scan—confirmed the diagnosis in 11 patients, in 14 patients diagnosis was supported by biopsy. No further data on making the diagnosis was available. Over the three time periods A, B, and C, the presence of vertebral fractures increased from 0.7 to 2.3% and 2.5%, without reaching statistical significance (*p* = 0.39).

### HLA-B27 subgroup analysis

HLA-B27 was positive in 48 of 314 analyzed patients (15.3%). The clinically noted distribution of bone lesions in HLA-B27-positive compared to HLA-B27-negative patients was comparable, except for a more common involvement of the calcaneus (27.5% vs 13.1%; *p* = 0.02), without related spinal or pelvic involvement. The metatarsal bone was also more commonly affected (20.0% vs 13.1%; *p* = 0.2) but did not reach a significant difference. Diagnostic criteria for enthesitis-related arthritis were fulfilled in 16/672 patients (2.4%) as described above. There was no evidence for a difference in the presence of arthritis in HLA-B27-negative or HLA-B27-positive patients. Seven of 266 HLA-B27-negative patients were diagnosed with ERA (2.9%) compared to 6 of 48 HLA-B 27-positive patients (14.6%) (*p* = 0.016). Radiologically, the distribution of lesions in HLA-B27-positive (*n* = 21 patients with imaging) compared to HLA-B27-negative patients (*n* = 130 with imaging) was quite comparable, with one exception: in more than half of the HLA-B27-positive patients (54.6%), at least one lesion in the bones of the foot (vs. 27.7% in HLA-B27-negative patients, *p* = 0.023) was described. The mean number of lesions was higher in HLA-B27-positive patients than in HLA-B27-negative patients when radiological lesions per patient were considered (mean/SD/median: 3.5/2.0/3.9 versus 2.7/2.0/2.2). However, the comparison did not reach significance. Of note, clinically, no significant difference in the mean number of lesions was reported in HLA-B27-positive versus HLA-B27-negative patients (*n* = 2.6 versus 2.3).

### Therapy

Almost all patients (96.9%) were treated with any medication during the time before the first documentation. Most of them (84.7%) received NSAIDs. Glucocorticoids were administered orally in a low-dose regimen (prednisone/prednisolone below 0.2 mg/kg/day) in 5.1% of patients and in 6.1% of patients at a dosage above 0.2 mg/kg/day, and overall steroid usage was noted in 9.6% of patients. Of interest, the higher the number of bone lesions was, the more glucocorticoids (up to 20% of patients) and biological DMARDs were used. Only 10.8% of the patients (71/657 patients) received DMARDs, and most of them were treated with methotrexate (4.6%) or sulfasalazine (4.1%). Biological agents included etanercept (*n* = 11; 1.7%), adalimumab (*n* = 5), or certolizumab (one patient). Bisphosphonates were administered in 6.1% of the patients. Thirty-four percent of those had vertebral fractures (for comparison of the therapeutic strategies in different cohorts, see additional file 2).

Of interest, DMARD use in HLA-B27-positive patients was higher than that in HLA-B27-negative patients (22.7% versus 8.3% of patients (*p* = 0.004)). In particular, MTX (9.1% versus 2.1%; *p* = 0.01) and sulfasalazine (13.6% versus 3.7%; *p* = 0.01) were used more often in HLA-B27-positive patients. In the different time periods A-B-C, the use of NSAIDs decreased significantly (90%, 87.3%, and 80.6% (*p* = 0.001)). The use of glucocorticoids did not change over time. Bisphosphonate use increased from 4.0% (A) to 6.9% (C). This change, however, was not statistically significant (*p* = 0.45). Methotrexate usage increased from 1.5% (A) to 5.6% (C) (*p* = 0.14), whereas sulfasalazine was used less often (5.1%, 4.2%, 3.6%). Etanercept use increased in low numbers from 0 to 1.9% and 2.3% (*p* = 0.21).

### Outcome parameters including the PedCNO score in the first year of follow-up

We calculated the PedCNO score in 186 patients. In this group, information on the baseline and at the 1-year follow-up was available for the score. A score of 30% improvement was reached by 59.1% of patients after 1 year (PedCNO30). PedCNO50 was reached by 54.8% of the patients, and 43% of patients reached 70% improvement after 1 year (PedCNO70). Considering radiological remission with a lesional number of zero, 17% of patients were in remission at the 1-year follow-up, and 36.7% had no pain.

In this patient group, 86.5% received NSAIDs at baseline and 69.6% at follow-up. No DMARD usage was noted in 65.8%/56.7%, respectively. Conventional DMARDs were used in 10.1%/18.5%, and biological DMARDS was used in 1.3%/5.1%. At the current state of analysis, a long-term therapeutic effect can only be estimated. This question will be addressed in the analysis of the long-term cohort, which has been started already.

## Discussion

The current analysis of NPRD, including the years 2009 to 2018, comprises the largest CNO patient documentation ever reported in the literature. Compared to the largest cohort thus far in the Eurofever registry and other relevant national cohorts, almost 800 patients have been analyzed for their clinical description, radiological imaging, laboratory features, and potential risk factors already emerging over the first 12 months of initial follow-up (Table 2, modified from [27]).

**Table 2:** Characteristics of patients reported in selected CNO reference cohorts

	Canada 2002 [23]	USA 2012 [24]	France 2015 [25]	UK 2016 [10]	Germany 2015 [26]	Eurofever 2017 [27]	Germany NPRD 2020
Number of Patients	23	70	178	41	95	486	774
Male %	17	33	31	25	42	36	37
Female %	83	67	69	75	56	64	63
Mean of age at disease onset	9	9.7	10.9	9	11.7	9.9	11.1
Delay of diagnosis in months	13	6	17	15	11	12	8.4
Number of lesions mean	4	3.5	3	N/A	4	4	3
Unifocal in %	43	29	7	24	16	29	37
Multifocal in %	57	71	93	76	84	71	63
Active disease in % after follow-up	22	39	66	N/A	33	50	47
Months of follow-up	68	22	48	N/A	49	N/A	12

*NPRD* National Pediatric Rheumatologic Database, *N/A* not available

The mean age of 11 years at disease onset in affected individuals and the predominance of females are comparable to other larger cohorts in the literature [6, 10, 23–31]. In addition, the lesional bone distribution was comparable, particularly the involvement of the pelvis, spine, and lower extremities. With regard to the patients’ reported bone involvement, TIRM-STIR MRI analysis showed a higher number of lesional involvement than clinical diagnosis. In particular, more spinal lesions and lesions of the pelvis and of the lower extremities were identified by MRI (Fig. 2). Higher MRI sensitivity compared to bone scintigraphy has already been reported in the literature [11]. Over the three different time periods, the parameters of *initial disease activity*, *patients’ noted pain,* and *general well-being* worsened to some extent. In Germany and Europe, awareness of CNO as a disease entity has improved in the last 10 years. Concomitant or associated pain syndromes came into focus, maybe causing a change in reporting characteristics over time?

For the first time in the literature, we were able to report an ongoing lower initial body height, weight, and BMI (kg/m^2^) in CNO patients compared to a standardized, age-matched national cohort (Fig. 4A, B). Since this finding is already present at disease onset, it does not seem to be associated with modes or sequelae of therapy but may be a disease-specific factor of bone development or a consequence of chronic bone inflammation. While laboratory parameters only showed limited signs of systemic inflammation (CRP was elevated in 18% of patients), this growth/stature delay may be an important factor in future research. Of note, since there was a significant delay of diagnosis of approximately 4 months in our cohort (comparable to previous reports), the improvement of early CNO diagnosis should be an important goal.

The current cohort can be considered “representative” and without a reporting bias of severely affected long-term patients, in part because the NPRD cohort records data from all German pediatric rheumatology centers. In this regard, we observed that the mean number of clinical (*n* = 2.3) or radiological lesions (*n* = 2.7) was lower than that in comparable cohorts. In the Eurofever cohort [27], the mean number of lesions was 4.1 defined by MRI, 3.5 defined by bone scintigraphy, and 1.9 defined by X-rays [27]. As most patients underwent MRI diagnostics (648 of 774 patients), the mean number of reported lesions of 2.7 may have reflected overall lower disease activity. In addition, the visual analog scale VAS (range 0–10) describing disease activity and well-being, as reported by physicians and patients, was lower than in the local German Cohort reported by Beck et al. in 2010 (mean pain VAS initially was 4.4, the mean C-HAQ score (range 0–3) was 0.75 and the mean overall well-being VAS was 5.0 at the initial visit at study entrance/diagnosis) [18]. VAS and NRS show comparable results [32]. The Beck study is a prospective cohort documenting the effectiveness of NSAIDs by patients’ and physicians’ reported outcomes introducing the PedCNO score. The PedCNO score was compared to that of this previous cohort; we found 59.1%/54.8%/43% of the patients in PedCNO30/50/70. The improvement reached was comparable but lower than that reported by Beck et al. [18]. They described 62%/57%/54% in PedCNO30/50/70 score levels [18]. Of note, the time of data collection was not precisely the same as in the prospectively controlled cohort by Beck et al. There, the PedCNO score was comparing first presentation and 1-year follow-up. While the NPRD documents the patient outcome once at any time of presentation throughout the year, the currently described outcome data do not precisely reflect the initial situation at diagnosis without therapy: the first documentation in the registry was done at a mean of 8.4 months after disease onset and approximately 4 months after the start of rheumatology care/treatment.

Of note, the mean number of MRI-defined bone lesions was usually a stable feature of CNO disease throughout the first 6 months of therapy, while the patient’s and physician’s global outcomes may have improved soon after treatment initiation [18]. Thus, the PedCNO score seems to be a useful tool for comparing treatment efficacy. Of note, the PedCNO score has been implemented with its composing items in the current international effort to establish “treat to target” protocols [16].

The role of HLA-B27 as a marker of CNO disease per se and of disease activity has been debated for decades. HLA-B27-positive patients showed higher numbers of lesions clinically as well as radiologically, with a particular involvement of six or more bones. The prevalence of HLA-B27 was 15.3%, which is somewhat higher than the regional prevalence in Germany of approximately 10% and higher than that in Eurofever (7.4%) [27], the cohorts reported by Beck et al. (8%) [18] and by Wipff et al. (7%) [25]. A total of 14.6% of HLA-B27-positive CNO patients, compared to 2.4% of the whole cohort, were diagnosed with enthesitis-related arthritis. Therefore, the HLA-B27 presence may be a prognostic marker for the development or codiagnosis of ERA. HLA-B27-positive CNO patients had a significantly higher involvement of the tarsal bones, including the calcaneus. Although the diagnosis of ankylosing spondylitis was not reported in our cohort, multifocal chronic bone inflammation (CRMO subtype) affecting the tarsal bones in addition to the presence of HLA-B27 may be a significant risk factor for the development of spondyloarthropathy (SPA). In contrast, Vittecoq et al. described SPA evolution in a small French cohort without the presence of HLA-B27 [5].

The use of NSAIDs during the first year of follow-up was as high as that reported in other cohorts. The use of conventional DMARDs, mainly methotrexate and sulfasalazine, was, however, limited to 10.8% of the patients. Only a few patients were treated with TNF-blocking agents. We searched for positive predictors at disease onset associated with aggravation of disease or resistance to NSAID treatment alone leading to the use of DMARDs. Interestingly, patients who were HLA-B27-positive revealed a DMARD use of 22.7% compared to HLA-B27-negative patients (8.3%), suggesting higher disease activity. In particular, methotrexate and sulfasalazine were used for disease-modifying therapy in HLA-B27-positive patients. The higher the lesional number was noted, the more glucocorticoid use was reported in addition to a moderate increase in the use of biological DMARDs.

Overall glucocorticoid use did not change over the years of inclusion; however, bisphosphonate use increased to 6.9% of patients, associated with higher axial/spinal involvement over time. More sacroiliitis but less arthritis was reported. Fewer biopsies were performed over time; however, these samples were subjected to more molecular microbial analysis, including mycobacterial and 16S rRNA eubacterial PCR.

The current CARRA CNO treatment protocol includes a treatment plan for the first 12 months of the disease course in patients refractory to NSAIDs or patients with primary spinal involvement [11]. Our cohort shows that medications implemented in CARRA “treatment to target” recommendations have been used in daily routine. It will be of particular interest to compare future treatment outcome data with this current prospective long-term national cohort. Long-term follow-up analysis in this cohort is planned to be reported consecutively.

## Conclusions

This large cohort of CNO patients shows that pediatric CNO patients are at risk for lower weight and height, implying an overall energy disbalance and showing the necessity for early diagnosis and treatment. As new biomarkers in daily routine are still lacking, the data imply that HLA-B27 might be a potential risk factor for a severe disease course. Treatment response was favorable in the first year of disease according to the data in the German database, and the general disease course tended to be rather mild.

### Supplementary Information

**Additional file 1:** Age distribution at disease onset (PPTX 38 kb)

**Additional file 2:** NSAID and DMARD therapy in selected CNO cohorts. NPRD: National Pediatric Rheumatologic Database (PPTX 43 kb)

## Data Availability

The datasets used and/or analyzed during the current study are available from the corresponding author on reasonable request.

## Appendix

List of centers participating in the German NPRD.

Thomas Berger, Vestische Kinder- und Jugendklinik Datteln, Rheumatologie/Immunologie, Datteln; Rainer Berendes, Kinderklinik St. Marien, Landshut; Regine Borchers, Universitätsklinikum Augsburg, Klinik für Kinder- und Jugendliche, Augsburg; Michael Borte, Städtisches Klinikum St. Georg, Klinik für Kinder- und Jugendmedizin, Leipzig; Jürgen Brunner, Medizinische Universität Innsbruck, Kinder- und Jugendheilkunde, Innsbruck; Frank Dressler, Medizinische Hochschule Hannover, Kinderklinik, Hannover; Ivan Foeldvari, Hamburger Zentrum für Kinder- und Jugendrheumatologie, Schwerpunktpraxis am Klinikum Eilbek, Hamburg; Dirk Föll, Universitätsklinik Münster, Klinik für Pädiatrische Rheumatologie und Immunologie, Münster; Anja Fröhlich, Universitätsklinik Eppendorf, Klinik und Poliklinik für Kinder- und Jugendmedizin, Hamburg; Matthias Galiano, Universitätsklinikum Erlangen, Kinder- und Jugendklinik, Erlangen; Hermann Girschick, Vivantes Klinikum Friedrichshain, Berlin; Jürgen Grulich-Henn, Universitätsklinikum Heidelberg, Zentrum für Kinder- und Jugendmedizin - Kinderheilkunde I, Heidelberg; Johannes-Peter Haas, Deutsches Zentrum für Kinder- und Jugendrheumatologie, Garmisch-Partenkirchen; Maria Haller, Kinderarztpraxis, Gundelfingen; Georg Heubner, Städtisches Klinikum Dresden-Neustadt, Klinik für Kinder- und Jugendmedizin, Dresden; Nadja Hofmann, Sozialstiftung Bamberg, Klinik für Kinder und Jugendliche, Bamberg; Anette Holl-Wieden, Universitätsklinikum Würzburg, Kinderklinik und Poliklinik, Würzburg; Gerd Horneff, Asklepios Kinderklinik St. Augustin, Zentrum für Allgemeine Pädiatrie und Neonatologie, Sankt Augustin; Anton Hospach, Zentrum für Pädiatrie, Olgahospital, Klinikum Stuttgart; Regina Hühn, Martin-Luther-Universität Halle-Wittenberg, Halle (Saale); Markus Hufnagel, Zentrum für Kinder- und Jugendmedizin, Universitätsklinikum, Freiburg; Ales Janda, Universitätsklinikum Ulm, Klinik für Kinder- und Jugendmedizin, Ulm; Annette Jansson, Dr.-von-Haunersches Kinderspital der LMU, Kinderklinik und Kinderpoliklinik, München; Tilmann Kallinich, Universitätsmedizin Berlin - Charité, Campus Virchow-Klinikum, Otto-Heubner-Centrum für Kinder- und Jugendmedizin, Berlin; Thomas Keller, Josefinum Krankenhaus, Klinik für Kinder und Jugendliche, Augsburg; Hans Kössel, Klinikum Westbrandenburg, Kinder- und Jugendmedizin, Brandenburg; Elke Lainka, Universitäts-Kinderklinik Essen, Zentrum für Kinder- und Jugendmedizin, Essen; Georg Leipold, Gemeinschaftspraxis Kinder- und Jugendärzte, Regensburg; Jan Maier, Kinderarztpraxis, Leinfelden-Echterdingen; Kristina Mathony, Städtisches Klinikum Dessau, Klinik für Kinder- und Jugendmedizin, Dessau; Almut Meyer-Bahlburg, Universitätsmedizin Greifswald, KöR, Abt. Allgemeine Pädiatrie, Greifswald; Kirsten Minden, Universitätsmedizin Berlin - Charité, Campus Virchow-Klinikum, Otto-Heubner-Centrum für Kinder- und Jugendmedizin, Berlin; Kirsten Mönkemöller, Kinderkrankenhaus der Stadt Köln, Kinder- und Jugendmedizin, Köln; Tim Niehues, Helios Klinikum Krefeld, Pädiatrische Institutsambulanz, Krefeld; Nils Onken, Kinderarztpraxis, Lüneburg; Prassad Oommen, Med. Einrichtungen der Heinrich-Heine-Universität, Zentrum für Kinder -und Jugendmedizin, Düsseldorf; Claudia Präger, Diakonie-Klinikum Schwäbisch Hall, Kinderklinik, Schwäbisch Hall; Jürgen Quietzsch, DRK Krankenhaus Lichtenstein, Klinik für Kinder- und Jugendmedizin, Lichtenstein; Christiane Reiser, Kinderklinik, Landeskrankenhaus Bregenz, Bregenz; Christoph Rietschel, Clementine Kinderhospital, Klinik für Kinder- und Jugendmedizin, Frankfurt; Betina Rogalski, Kinderrheumatologische Privatpraxis, Alsbach-Hähnlein; Michael Rühlmann, Kinderarztpraxis, Göttingen; Peggy Rühmer, Helios Vogtland-Klinikum Plauen, Fachambulanz der Klinik für Kinder- und Jugendmedizin, Plauen; Axel Sauerbrey, Helios Klinikum Erfurt, Klinik für Kinder- und Jugendmedizin, Erfurt; Anja Schnabel, Universitätsklinikum Carl Gustav Carus, Klinik und Poliklinik für Kinder- und Jugendmedizin, Dresden; Martin Scholten, Universitätsklinikum Jena, Klinik für Kinder- und Jugendmedizin, Jena; Volker Schuster, Universitätsklinik und Poliklinik für Kinder und Jugendliche, Rheumaambulanz, Leipzig; Catharina Schütz, Universitätsklinikum Carl Gustav Carus, Klinik und Poliklinik für Kinder- und Jugendmedizin, Dresden; Anja Sonnenschein, Johann-Gutenberg-Universität Mainz, Zentrum für Kinder- und Jugendmedizin, Mainz; Claudia Stollbrink, Universitätsklinikum Aachen; Kinderklinik RWTH Aachen, Klinik für Kinder- und Jugendmedizin, Aachen; Ralf Trauzeddel, Helios Klinikum Berlin-Buch, Klinik für Kinder- und Jugendmedizin, Berlin; Philipp von Bismarck, Universitätsklinikum Schleswig Holstein - Campus Kiel, Klinik für Kinder- und Jugendmedizin, Kiel; Frank Weller-Heinemann, Klinikum Bremen Mitte - Professor-Hess-Kinderklinik, Zentrum für Kinder- und Jugendrheumatologie, Bremen; Daniel Windschall, St. Josef-Stift Sendenhorst, Abt. Kinder- und Jugendrheumatologie, Sendenhorst

## Abbreviations

BMI: Body mass index
CARRA: Childhood Arthritis and Rheumatology Research Alliance
C-HAQ: Childhood Health Assessment Questionnaire
CNO: Chronic nonbacterial osteomyelitis
CRMO: Chronic recurrent multifocal osteomyelitis
CRP: C-reactive protein
CTPs: Consensus treatment plans
DMARDs: Disease-modifying anti-rheumatic drugs
ESR: Erythrocyte sedimentation rate
ERA: Enthesitis-related arthritis
HLA: Human leukocyte antigen
ILAR: International League Against Rheumatism
MRI: Magnetic resonance imaging
MTX: Methotrexate
NPRD: National Pediatric Rheumatologic Database
NRS: Numeric rating scale
NSAIDs: Nonsteroidal anti-inflammatory drugs
PedCNO: Pediatric chronic nonbacterial osteomyelitis follow-up treatment score
QoL: Quality of life
SAPHO: Synovitis acne pustulosis hyperostosis osteitis syndrome
SPA: Spondyloarthropathy
STD: Standard deviation
STIR: Short tau inversion recovery
T2T: Treat-to-target recommendations
TIRM: Turbo inversion recovery measurement
TNF: Tumor necrosis factor
