# Supplementary figures and images for: First-year follow-up of children with chronic nonbacterial osteomyelitis—an analysis of the German National Pediatric Rheumatologic Database from 2009 to 2018
Source: Arthritis Res Ther. 2021 Nov 8;23:281. doi: 10.1186/s13075-021-02658-w (PMC8573927; doi:10.1186/s13075-021-02658-w)

## Slide 1
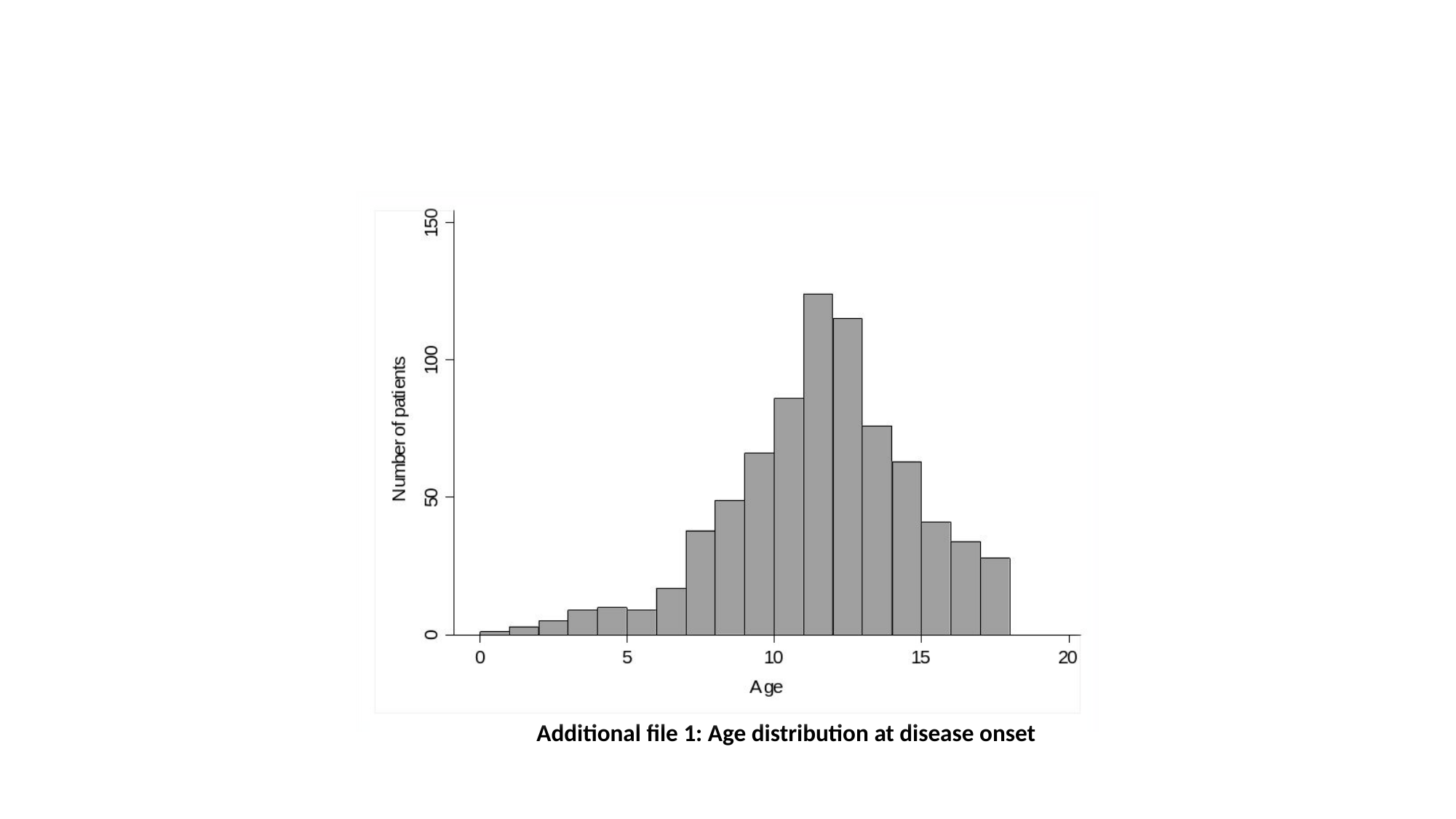

# Additional file 1: Age distribution at disease onset

Supplement: Supplementary file 1 — Additional file 1: Age distribution at disease onset (PPTX 38 kb) [file 13075_2021_2658_MOESM1_ESM.pptx]
