# Supplementary material for: First-year follow-up of children with chronic nonbacterial osteomyelitis—an analysis of the German National Pediatric Rheumatologic Database from 2009 to 2018
Source: Arthritis Res Ther. 2021 Nov 8;23:281. doi: 10.1186/s13075-021-02658-w (PMC8573927; doi:10.1186/s13075-021-02658-w)
Supplement: Supplementary file 2 — Additional file 2: NSAID and DMARD therapy in selected CNO cohorts. NPRD: National Pediatric Rheumatologic Database (PPTX 43 kb) [file 13075_2021_2658_MOESM2_ESM.pptx]

## Slide 1
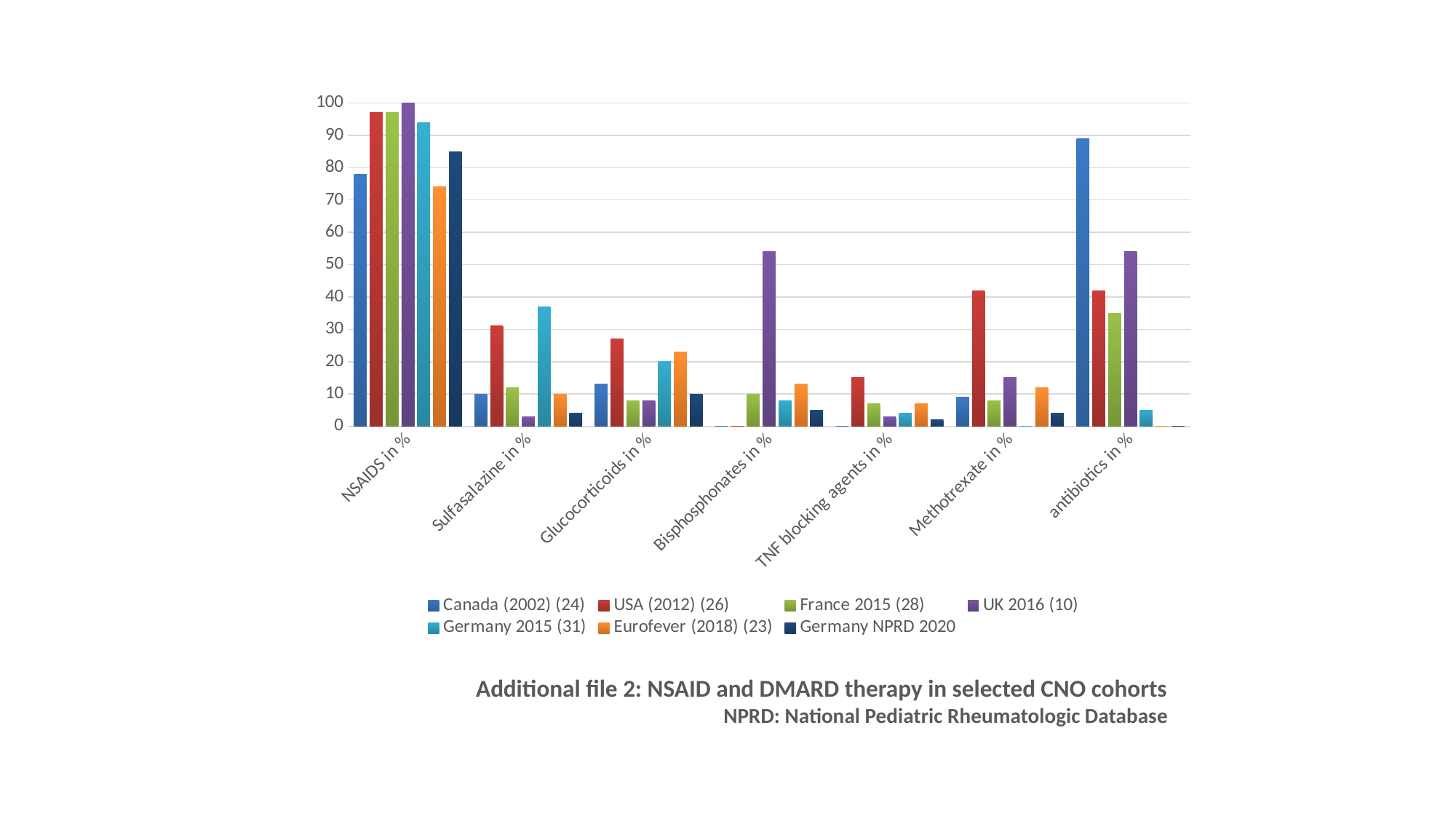

### Chart:
| Category | Canada (2002) (24) | USA (2012) (26) | France 2015 (28) | UK 2016 (10) | Germany 2015 (31) | Eurofever (2018) (23) | Germany NPRD 2020 |
|---|---|---|---|---|---|---|---|
| NSAIDS in % | 78.0 | 97.0 | 97.0 | 100.0 | 94.0 | 74.0 | 85.0 |
| Sulfasalazine in % | 10.0 | 31.0 | 12.0 | 3.0 | 37.0 | 10.0 | 4.0 |
| Glucocorticoids in % | 13.0 | 27.0 | 8.0 | 8.0 | 20.0 | 23.0 | 10.0 |
| Bisphosphonates in % | 0.0 | 0.0 | 10.0 | 54.0 | 8.0 | 13.0 | 5.0 |
| TNF blocking agents in % | 0.0 | 15.0 | 7.0 | 3.0 | 4.0 | 7.0 | 2.0 |
| Methotrexate in % | 9.0 | 42.0 | 8.0 | 15.0 | 0.0 | 12.0 | 4.0 |
| antibiotics in % | 89.0 | 42.0 | 35.0 | 54.0 | 5.0 | 0.0 | 0.0 |Additional file 2: NSAID and DMARD therapy in selected CNO cohorts
NPRD: National Pediatric Rheumatologic Database
